# A multidisciplinary approach to tackling invasive species: barcoding, morphology, and metataxonomy of the leafhopper *Arboridia adanae*

**DOI:** 10.1038/s41598-023-49410-9

**Published:** 2024-01-26

**Authors:** Riccardo Piccinno, Alessia Tatti, Sabina Avosani, Giulio Galla, Valentina Lazazzara, Federico Pedrazzoli, Nicola Zadra, Mirco Rodeghiero, Gabrijel Seljak, İnanç Özgen, Heidi C. Hauffe, Vincenzo Verrastro, Marco Valerio Rossi Stacconi, Valerio Mazzoni, Omar Rota-Stabelli

**Affiliations:** 1 Center Agriculture Food Environment (C3A), University of Trento, San Michele All’Adige, Trento, Italy; 2 Plant Protection Unit, Research and Innovation Centre, San Michele All’Adige, Fondazione Edmund Mach, Trento, Italy; 3 Department of Biology and Biotechnology “L. Spallanzani”, University of Pavia, Pavia, Italy; 4 Scuola Universitaria Superiore IUSS Pavia, Pavia, Italy; 5 Faculty of Science and Medicine, University of Fribourg, Fribourg, Switzerland; 6 Conservation Genomics Research Unit, Research and Innovation Centre, Fondazione Edmund Mach, San Michele All’Adige, Trento, Italy; 7 Institute for Sustainable Plant Protection, National Research Council of Italy, Sesto Fiorentino, Florence, Italy; 8 National Biodiversity Future Center (NBFC), S.c.a.r.l., Palermo, Italy; 9 Kromberška Cesta 8, 5000 Nova Gorica, Slovenia; 10 Bioengineering Department, Engineering Faculty, Fırat University, Elazığ, Turkey; 11 International Centre for Advanced Mediterranean Agronomic Studies (CIHEAM) - Bari, Valenzano, Bari, Italy

**Keywords:** Molecular evolution, Phylogenetics, Invasive species, Microbiome, Classification and taxonomy, Entomology

## Abstract

The leafhopper genus *Arboridia* includes several species that feed on *Vitis vinifera* and cause leaf chlorosis. We report the first alien *Arboridia* infestation in Italy in 2021 in an Apulian vineyard. To confirm the taxonomic status of the species responsible for crop damage, and reconstruct its demographic history, we barcoded individuals from Apulia together with *Arboridia* spp. from Crete (Greece), *A. adanae* from Central Turkey and other specimens of the presumed sister species, *A. dalmatina* from Dalmatia (Croatia). Molecular phylogenies and barcoding gap analysis identified clades not associated with sampling locations. This result is incongruent with classical specimen assignment and is further supported by morphological analyses, which did not reveal significant differences among the populations. Therefore, we propose *A. dalmatina* as a junior synonym of *A. adanae*, which would become the only grapevine-related *Arboridia* species in the eastern Mediterranean. To further characterise *A. adanae* evolution, we performed a molecular clock analysis that suggested a radiation during the Pleistocene glaciations. Finally, to assess whether the Apulian individuals carried microorganisms of agricultural relevance, we sequenced their bacterial microbiota using 16S rRNA amplicon sequencing identifying three phytopathogens not generally associated with *Arboridia* activities as well as *Wolbachia* in one Apulian haplogroup. We discuss the agricultural implications of this infestation.

## Introduction

The increasing number of invasive species detected globally represents a serious ecological problem. For example, the presence of alien species alters ecosystem function and, in the most serious cases, leads to autochthonous species extinction. When the invasive species is of agricultural interest, it can also represent a threat to the local economy^1^. This is the case of several leafhopper species (Hemiptera: Cicadellidae), such as the North American *Erasmoneura vulnerata* Fitch, 1851,^2^ and the African *Jacobiasca lybica* Bergevin & Zanon, 1922^3^, which are known to cause damage to various crops, in particular grapevine.

The genus *Arbordia* Zakhvatkin, 1946 (Cicadellidae: Typhlocibinae), includes more than 80 leafhopper species^4^ that have been found in Europe, Asia and North America^5,6^. Of them, at least five species *(A. kakogawana* Matsumura, 1932, *A. vinealis* Ahmed, 1970, *A. kermanshah* Dlabola, 1963, *A. adanae* Dlabola, 1957, and *A. dalmatina* Wagner, 1962) are reported to use grapevine as their main host plant. *Arboridia* species are mesophyll-feeders^7–10^: adults and nymphs feed directly on leaves, causing stippling due to the piercing and sucking activity on the lower side of leaves. Therefore, they are unlikely to transmit phytoplasmas^11^, but can cause severe loss of chlorophyll, which eventually leads to chlorosis and related yield reduction^9^. Few studies have investigated the evolution and origin of *Arboridia*, which, according to mitogenomic analysis, was estimated to have diverged from the genera *Erythroneura* Fitch, 1851, and *Eratoneura* Young, 1952, about 36 million years ago (MYA)^12^.

In summer 2021, an infestation of leafhoppers associated with diffuse leaf stippling was recorded in a vineyard in southern Italy, near Valenzano, in the region of Apulia. Before this date, no grapevine *Arboridia* species had ever been reported from the Italian peninsula. The collected specimens were preliminarily identified by us as *Arboridia* spp. based on the morphological analysis of macro and microscopic characters^13,14^. We used this opportunity to shed light on this new Italian invasive species and discuss possible invasion consequences.

The two grapevine-related *Arboridia* species distributed newarest to Apulia are *A. dalmatina* (from the Balkans) and *A. adanae* (Anatolia), but their biology, ecology, and evolution are poorly known. Since these species might represent a threat for vineyards in Italian and other Mediterranean regions, further knowledge of the taxonomy and microbiota of these species is required to track its invasion routes and to identify microorganisms of potentially pathogenetic and/or control relevance. Therefore, here we performed molecular phylogenetic analyses and confirmed our results using traditional morphological classification, followed by amplicon sequencing was used to characterise the microbiota of the same specimens. In addition, we estimated the radiation of the interested species to gain insights on the evolution of this genus. Overall, this multidisciplinary approach was an efficient method for characterising the phylogeny, taxonomy, and evolution of an agriculturally relevant invasive insect species.

## Materials and methods

### Sample collection

*Arboridia* individuals were collected in vineyards with nets and stored in 80% ethanol (v/v): 15 from Valenzano (Apulian: Apulia, Italy, 41°03′N, 16°52′E; 23/08/2021), 16 from Potomje (Dalmatian: Dalmatia, Croatia, 42°56′N, 17°18′E; October 2021; purportedly *A. dalmatina*), 29 from Yurtbaşı (Turkish: Elazığ, Turkey, 38°35′N, 28°48′E; 13/10/2022 purportedly *A. adanae*) and five from Kapariana (Cretan: Heraclion, Greece, 35°03′N, 24°53′E; 27/4/2021; location between the Balkans and Anatolia).

### Phylogenetic, barcoding gap and molecular clock analyses

In order to resolve taxonomic questions from the molecular perspective and test phylogenetic hypotheses, a total of 23 individuals were sequenced (five each from Apulia, Crete, and Dalmatia, and eight from Turkey). Total DNA was purified from lyophilised and homogenised individuals with the NucleoSpin Tissue kit (MACHEREY–NAGEL GmbH & Co. KG), according to manufacturer’s instructions. The purified DNA was eluted in 30 μl Buffer BE.

The cytochrome oxidase subunit I (COI) gene was amplified using the universal primers LCO1490 (5'-GGTCAACAAATCATAAAGATATTGG-3') and HCO2198 (5'-TAAACTTCAGGGTGACCAAAAAATCA-3')^15^ to a final concentration of 0.3 μM each, the GoTaq® Green Master Mix (Promega Corporation, CITY, USA), and 5 ng of DNA (2.5 ng/μl). Negative amplification controls (reactions carrying no DNA template) were included in each amplification process. The polymerase chain reaction (PCR) conditions were: 2 min at 95 °C, 5 × (45 s at 95 °C, 45 s at 45 °C, 1 min at 72 °C), 35 × (45 s at 95 °C, 45 s at 50 °C, 1 min at 72 °C), 5 min at 72 °C (modified from EPPO 2021). PCR products, after purification with Illustra ExoProStar1-Step (GE Healthcare, Little Chalfont, UK), were sequenced with the BigDye Terminator v3.1 cycle sequencing kit (Applied Biosystems, Foster City, CA, USA) on an Applied Biosystems 3130 xl Genetic Analyzer (Carlsbad, CA, USA) at the Sequencing and Genotyping Platform, Fondazione E. Mach (San Michele all’Adige, Italy).

The sequenced COI regions were visualised using Chromas software (Technelysium Pty Ltd). Forward and reverse reads were assembled using a Biopython script^16^; assembled sequences were then uploaded on NCBI GenBank (accession numbers provided in Table S1). A total of 16 *Arboridia* COI sequences were downloaded from the NCBI nucleotide database, nine of them belonging to *A. kakogawana* and seven belonging to *A. maculifrons* Vilbaste, 1968 (Table S1). All sequences were aligned using MAFFT version 7^17,18^ and trimmed manually at the 3’ and 5’ ends in order to remove gaps and ensure the reliability of the phylogenetic result. The final dataset comprised 637 aligned base pairs (bp) from 39 samples. We used this dataset to infer phylogenies under a Maximum Likelihood (ML) framework with the freeware RAxML^19^ using a GTR + Gamma replacement model with branch robustness assessed with 1,000 bootstrap replicates. FigTree (version 1.4.4) was used for topology visualisation and figure preparation.

To analyse the COI barcoding gap, we used our *Arboridia* sequences and all the Erythroneurini COI sequences available from the NCBI nucleotide database in November 2022. Sequences were aligned using MAFFT version 7^17,18^ and a custom python script was used to trim the head and tail of the alignment to avoid gaps. The resulting alignment was composed of 2875 sequences and 442 bp. The newly obtained alignment was used to calculate the pairwise distance matrix using the DistanceCalculator class of the TreeConstruction module of Biopython^16^ with the ‘identity’ model. To visualise the distribution of genetic distances and obtain the barcoding gap plot, the intraspecific and interspecific genetic distances were plotted in a histogram using the matplotlib library, excluding those involving the species *A. dalmatina* and *A. adanae*. Outlier sequences were detected considering the first (q_1/4_), the third quartiles (q_3/4_), and the interquartile range (IQR) according to Eqs. (1) and (2) of the pairwise distances distributions.

1 $${q}_{1/4}-1.5\cdot IQR$$

2 $${q}_{3/4}+1.5\cdot IQR$$

The same definitions of intraspecific and interspecific distributions were applied to pairwise distances. Finally, the genetic distances between sequenced *Arboridia* specimens were processed, plotted on a histogram and highlighted by the same python script.

We also employed the same dataset used for the ML analysis in a Bayesian framework to estimate divergence times between species using BEAST2^20^. After model selection, we employed a birth and death model and a relaxed log-normal clock as a tree priors and set the tree topology to the ML topology. To calibrate the tree, we added eleven COI sequences of a species belonging to Dikraneurini (*Dikrella cruentata* Gillette, 1898), the closest tribe for which COI sequences and fossil calibration were available, as well as fourteen COI sequences belonging to *Mileewa* Distant, 1908, a genus belonging to Mileewini tribe and Mileewinae subfamily, the closest Cicadellidae subfamily for which COI sequences and fossil calibration were also available (Table S1). We calibrated the tree root using the fossil of a Dekraneurini gen. sp.^21^, according to Yan et al. (2022)^12^, to set the Dikraneurini-Erythoneurini split at 17.5–90 MYA using a normal distribution (mean 53.6 MYA, standard deviation 18.4), and the fossil of *Youngeawea bicolorata* (Mileewinae: Mileewini) to set the minimum divergence time between *Mileewa* and Typhlocybinae at 44 MYA^22^. The analysis was run for 100 million Markov chain Monte Carlo (MCMC) iterations, or until it reached convergence, sampling every 10,000 steps after a 10% initial burn-in. We used Tracer 1.7.1^23^ to visualise convergence, which was considered reached when all variables had an Effective Sample Size (ESS) > 200 and a bell-shaped posterior distribution. Substitution saturation was checked using DAMBE^24,25^ considering Xia's observed index of saturation^26^.

### Morphological analysis

Forty-two *Arboridia* individuals were collected from the same sampling locations as those used for molecular analyses: 21 from Turkey, 10 from Apulia, and 11 from Dalmatia. Abdomens were removed from specimens, soaked in KOH solution (10%), heated to boiling for a few seconds to dissolve soft tissues, washed in distilled water, and transferred to glycerin for further dissection and standard microscopy. Digital micrographs were taken using a LEICA S9i stereomicroscope with integrated HD camera (LEICA Inc., Wetzlar, Germany). Morphological characters associated with species were identified following previously published keys^13,14^. As for macroscopic features, the colour pattern of the face, vertex, pronotum, scutellum and forewings were noted. Differences in body size were estimated between populations and between individuals showing diffuse red chromatism by comparing mean head capsule width, hind tibia length and distance between the vertex and the tip of the scutellum. Measurements were taken using a LAS X Life Science Microscope software (LEICA Inc., Wetzlar, Germany). Photographs and drawings were modified with GIMP 2.10.12 software (GNU General Public License). Before morphological features between populations were compared, assumptions for normal distributions and homoscedasticity were tested^27,28^. In cases where these assumptions were respected, a parametric one-way ANOVA was performed, otherwise a non-parametric Kruskal–Wallis test^29^ was used. Data were analysed and visualised using Graphpad Prism software (GraphPad Software, Inc., La Jolla, CA, USA).

### Metataxonomics

We characterised the whole microbiota of 14 *Arboridia* individuals for which COI was available collected from three European locations (five each from Apulia and Dalmatia; four from Crete). On the Animal, Environmental and Antique DNA Platform at the Fondazione E. Mach, the 16S rRNA gene V3-V4 region was amplified from the whole body in reactions of 25 μl containing 1X the KAPA HiFi HS ReadyMix Buffer (Roche), and the primers 341F_ILL (5’-TCGTCGGCAGCGTCAGATGTGTATAAGAGACAGCCTACGGGNGGCWGCAG-3’) and 805R-2_ILL (5’-GTCTCGTGGGCTCGGAGATGTGTATAAGAGACAGGACTACNVGGGTWTCTAATCC-3’)^30,31^ anchored with the Illumina forward and reverse overhang adapters (https://support.illumina.com/documents/documentation/) to a final concentration of 0.3 μM each and 100 ng of DNA (50 ng/μl). PCR amplification controls (reactions with no DNA template) were included in each amplification process. The PCR conditions were 3 min at 95 °C, 35 × (30 s at 95 °C, 30 s at 55 °C, 90 min at 72 °C), 7 min at 72 °C, using a Veriti™ 96-Well Fast Thermal Cycler (Applied Biosystems, USA). Quality checks for amplification success and efficiency were performed by capillary electrophoresis using the QIAxcel Advanced System (QIAGEN). Bacterial amplicons were sequenced using Illumina MiSeq 2 × 300 bp with a minimum depth of 100,000 reads per sample, performed on the Sequencing and Genotyping Platform, Fondazione E. Mach.

CutAdapt^32^ was employed to remove adapters from the 16S V3-V4 reads. Subsequent analytical steps were performed in R version 4.1.2 software^33^. The DADA2 package^34^ was used to filter the reads by quality, remove errors, merge the forward and reverse reads, remove chimaeras, and assign the taxonomy to the resulting ASVs using the Silva v138 as reference database^35,36^. Decontam^37^ was used to remove contaminant sequences defined by the negative controls. The phyloseq package^38^ was used to compute abundance and richness plots and statistics.

## Results and discussion

### Origin of the Apulian invasion

**Figure 1:**
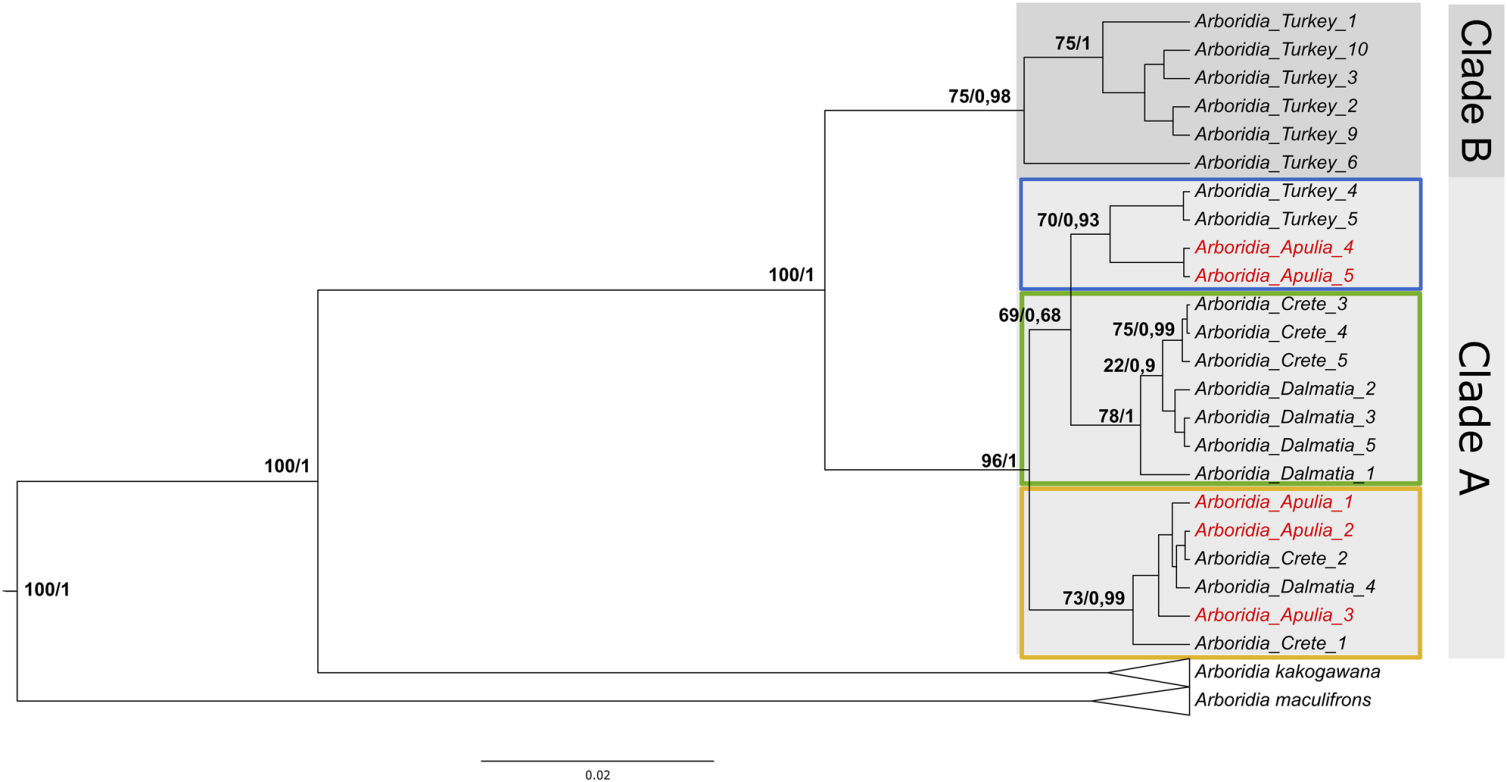
Phylogenetic tree of COI gene. In red sequences from the Apulian samples. The two numbers at the nodes are the number of bootstrap replicates under a ML framework and the posterior probability in a Bayesian framework, respectively. Supports were reported only for well-supported nodes considering both frameworks. Clade B (grey) is composed only of Turkish *Arboridia*, Clade A (light grey) is composed of Apulian, Cretan, Dalmatian and Turkish specimens. The three coloured squares define three genetic clusters in Clade A.

The COI phylogenetic tree (Fig. 1) suggested three clusters with high node support within Clade A: the ‘blue’ group composed of two Apulian samples (4 and 5) and two Turkish samples (4 and 5); the ‘green’ group composed of three Cretan samples (3, 4 and 5) and four Dalmatian samples (1, 2, 3 and 5); the ‘orange’ group composed of three Apulian samples (1, 2 and 3), two Cretan samples (1, 2) and one Dalmatian sample (4). The presence of individuals from different geographical locations in all three clusters indicated a complex geographic structure; in fact, the origin of the Apulian invasion cannot be identified from the COI sequences generated here. However, this result is compatible with a fragmented history of geographic isolation likely due to a recent spread of this species attributable to human activities, such as intensive Mediterranean trade related to viticulture. Wine- and viticulture-associated products have been traded across the Mediterranean basin as far back as 7000 B.C.^39^, therefore many sporadic gene flow events among the different leafhopper populations analysed might have occurred during the last 9000 years, leading to the complex pattern of genetic differentiation noted here.

The Apulian specimens belonged to two different groups in Clade A (‘blue’ and ‘orange’; Fig. 1), suggesting several possible evolutionary scenarios. For example, invasive *Arboridia* may be highly variable or there may have been more than one invasion event. Because *A. adanae* 4 and 5 from Turkey were genetically related to the Clade A, one invasion route may have been directly from Turkey. However, we cannot pinpoint the exact origin of additional invasions, since Apulian specimens 1, 2, and 3 were related to both Cretan and Dalmatian individuals.

### Molecular and morphological evidence of a unique Arboridia species in the Balkans and Turkey

As shown in the COI phylogenetic tree, two specimens, both originally assigned to *A. adanae* from Turkey (*A. adanae* 4 and 5), clustered with 100% support within *Arboridia*, but were in a separate cluster from the rest of Europe (Fig. 1). In particular, these two samples were closely related to two of the individuals sampled from Italy (*Arboridia* Apulia 4 and *Arboridia* Apulia 5) with ML support of 73/100 and posterior probability of 0.93. All specimens collected in Crete formed a cluster alongside Dalmatian *A. dalmatina* and Apulian *Arboridia*, indicating that three populations and Turkish 4 and 5 individuals belong to the same clade (Clade A, Fig. 1).

**Figure 2:**
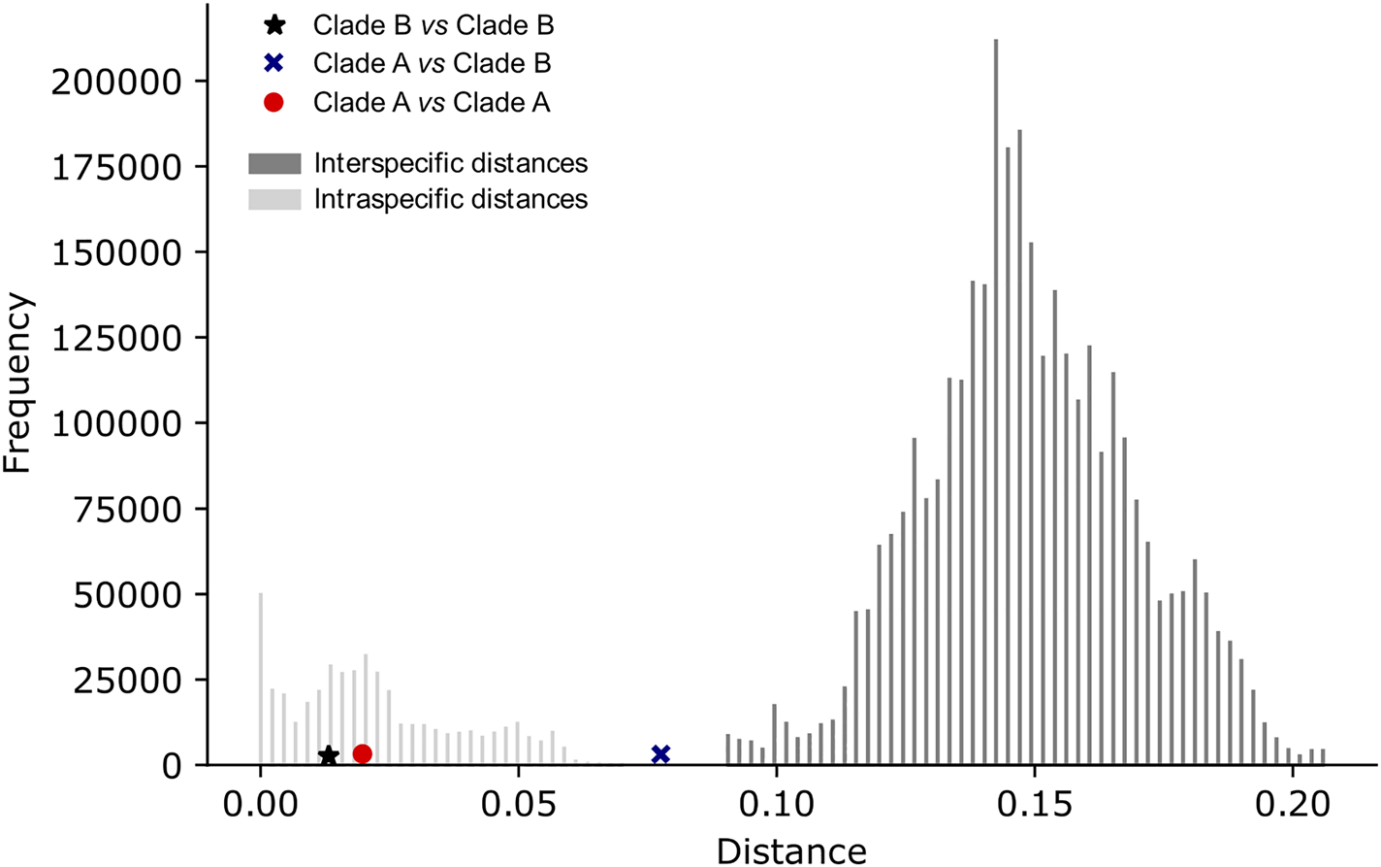
Barcoding gap of COI gene using Erythoneurini sequences. For Clade A, composed by Cretan, Dalmatian, Apulian individuals and Turkish specimens 4 and 5, the pairwise distance falls inside the set of intraspecific distances (red circle). In contrast, the pairwise distance of *A. adanae* samples 4 and 5 from Turkey and the rest of Turkish *A. adanae* (in Clade B) falls outside the intraspecific barcoding distance and inside the barcoding gap (blue cross).

These findings have raised doubts regarding the exact taxonomic status of *A. adanae* and *A. dalmatina*; these doubts were further substantiated by the barcoding gap analysis (Fig. 2). Whereas the distance among Clade A specimens fell within the distribution of intraspecific distances (Fig. 2 red circle), the distance between Clade A specimens and the rest of Turkish *Arboridia* (hereafter called Clade B, Fig. 1) fell inside the barcoding gap (Fig. 2, blue cross). These results indicate that we cannot taxonomically separate *A. dalmatina* from *A. adanae* on the basis of COI sequences.

**Figure 3:**
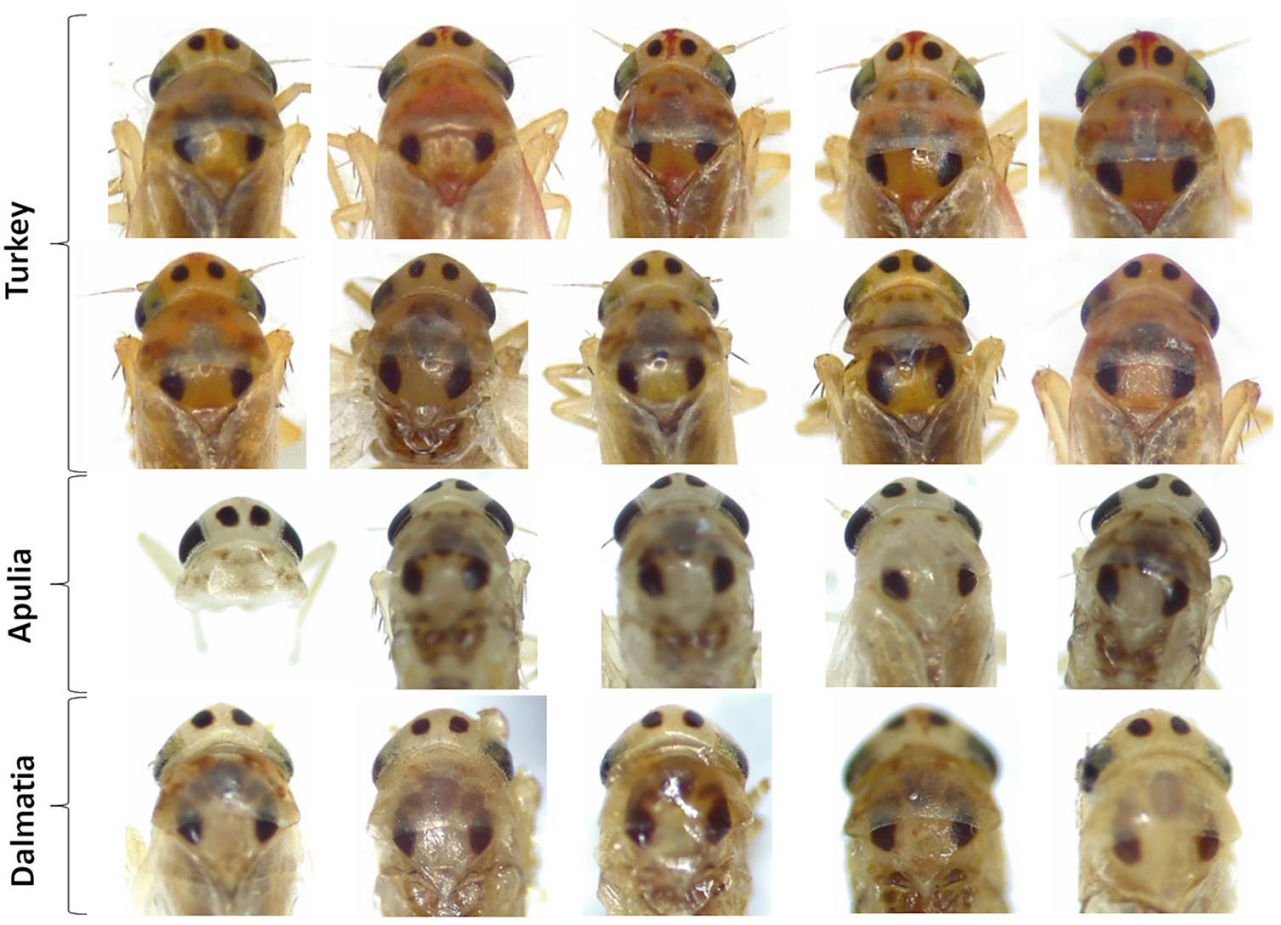
Dorsal view of the forebody (head and thorax) of *Arboridia* specimens collected from Turkey, Apulia and Dalmatia. In the top line, Turkish individuals show a clear colour polymorphism.

**Figure 4:**
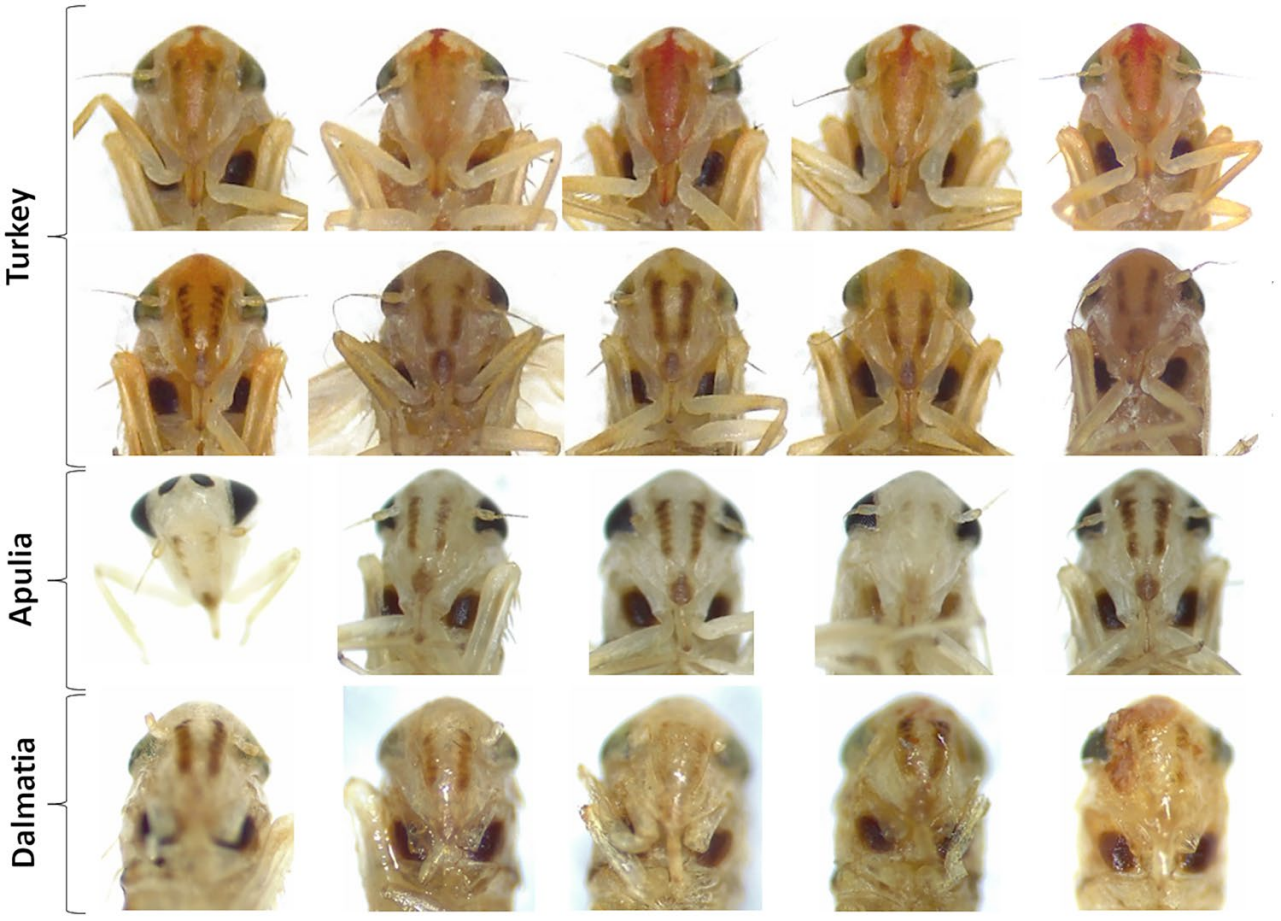
Ventral view of the forebody (head and thorax) of *Arboridia* specimens collected from Turkey, Apulia and Dalmatia. In the top line, Turkish individuals show a clear red chromatism on frons and postclypeus.

**Figure 5:**
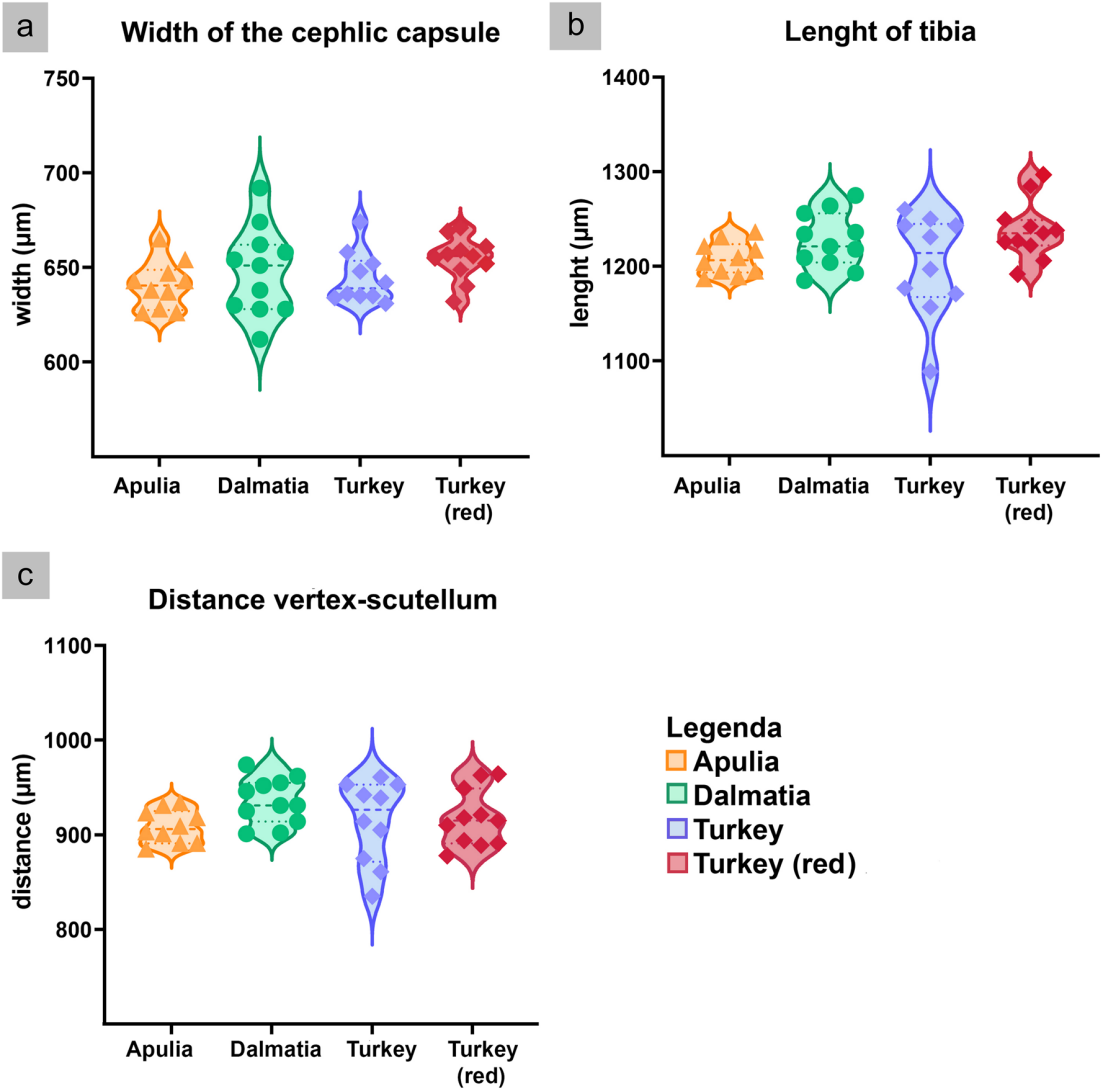
Violin plots of the morphological features considered here to compare the size of *Arboridia* specimens from the three sampling locations (10 Apulian specimens in orange, 11 Dalmatian specimens in green, 21 Turkish specimens differentiated in red with red pigment and in blue without the red pigment). (**a**) Analysis of the width of the cephalic capsule. (**b**) Analysis of the length of tibia. (**c**) Analysis of the distance between vertex and scutellum. No comparisons were statistically significant.

**Figure 6:**
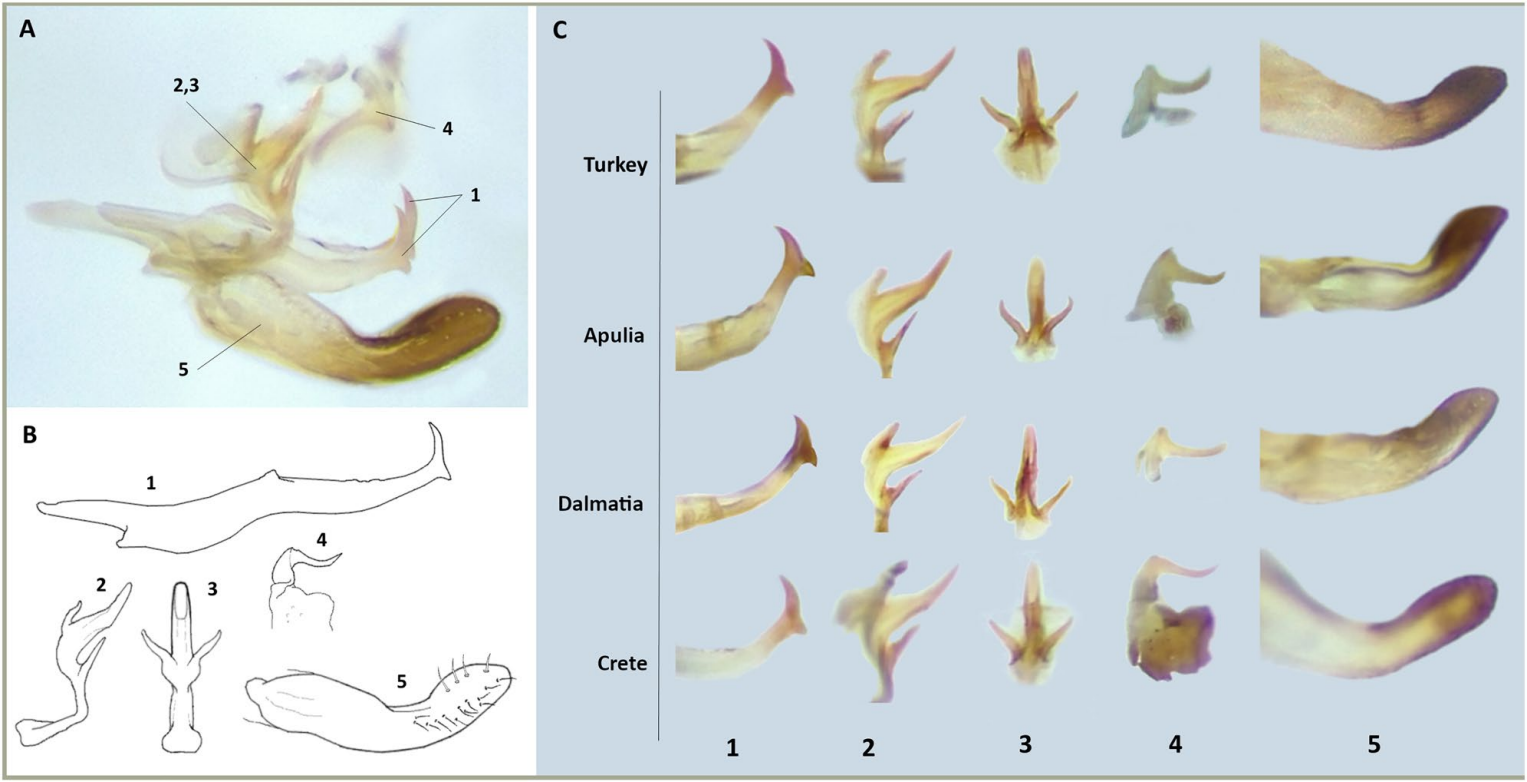
Male genitalia of the examined *Arboridia* did not show evident morphological differences. 1. style; 2. aedeagus, lateral view; 3. aedeagus, ventral view; 4. pygofer dorsal appendages; 5. subgenital plate. (**A**) General view of the male genitalia. (**B**) Schematic drawing of the different parts composing the male genitalia. (**C**) Micrographs of the different parts of the male genitalia showing comparison between the examined populations.

Results from morphological observations were also consistent with phylogenetic analysis. Body length of individuals (shown in Figs. 3, 4, 5) varied between 2.6 and 3.1 mm for females (average 2.72 ± 0.12 mm; n = 20) and between 2.4 and 2.9 for males (2.87 ± 0.12 mm; n = 21). Dorsally, adults were light to dark yellow with orange streaks running along the forewings, and dark brown tergites. Three prominent dark spots were present on the scutellum and other two on the vertex (Fig. 3). Smaller dark marks were present at the front of the pronotum. Ventrally, the legs were light yellow and sternites dark brown. Two brown stripes ran in parallel on each side of the postclipeus (Fig. 4). In some but not all of the Turkey specimens, a bright-red enlarged streak ran from the vertex to the anteclipeus, flanked by two whitish spots (Figs. 3 and 4). None of the morphological features measured were significantly different between populations (width of the cephalic capsule: One-Way Anova: F = 1.06, p = 0.35; distance vertex-scutellum: One-Way Anova: F = 2.45, p = 0.10; length tibia: Kruskal–Wallis test: H = 2.89, p = 0.24; Fig. 5). Similarly, male genitalia from all populations shared the same description: genital styles apically widened with a short ventral spur and a long, curved and pointed dorsal process (Fig. 6; n. 1). Aedeagus articulated to connective. In lateral view (Fig. 6; n. 2), the aedeagal shaft was markedly curved and apically tapered, with a long distal lobe at the apex. In ventral view (Fig. 6; n. 3), the aedeagus was symmetrical and straight, with distinct V-shaped processes placed basally, well-separated from the shaft, and shorter than the shaft. Connective fused with the aedeagus, with a short stem ending in an enlarged anterior lobe. Pygofer dorsal appendages (Fig. 6; n. 4) simple, movably articulated, and slightly curved in ventral view. Subgenital plates (Fig. 6; n. 5), in lateral view, slightly exceeded the pygofer, distally sclerotised and twisted, laterally bearing four to six macrosetae and numerous irregularly arranged short setae on the ventrolateral part of each plate. When comparing the genitalia of males belonging to the three sampling locations, no relevant morphological differences were observed at 50X magnification (Fig. 3C).

The reduced interspecific phylogenetic distance combined with the lack of significant morphological differences between Turkish and European *Arbordia* spp., means we cannot exclude either that the two species form a species complex with the possibility of interbreeding, or that these two species are actually subspecies of the same species, as was suggested by Dlabola (1963)^40^. Dlabola morphologically analysed both Turkish and Dalmatian specimens and considered Dalmatian specimens a subspecies of *A. adanae*, which he named *A. adanae vitisuga*^40^. In addition, the descriptions of genitalia published by Dlabola (1963)^40^ and Novak and Wagner (1962)^41^ are not distinct. Therefore, it is not clear why Dworakowska (1970)^42^ declared *A. adanae vitisuga* as a junior synonym of *A. dalmatina*. As for the body colour patterns, the red chromatism on the fore body (especially vertex and frons) of some Turkish specimens was not associated with distinct genetic or morphological traits and thus, it is likely that this variability is associated with insect phenology, a phenomenon that is well-known in the Erythroneurini tribe. For example, the grapevine leafhopper, *Zygina rhamni* Ferrari, 1882, widespread in the southern Mediterranean, is characterised by a variable pattern of large red markings and streaks found only on overwintering individuals, not those belonging to summer generations^43^. Such seasonal variability may also characterise *Arboridia*, although a phenological investigation would be required to clarify this aspect.

### The recent origin and divergence of the A. dalmatina-adanae complex

**Figure 7:**
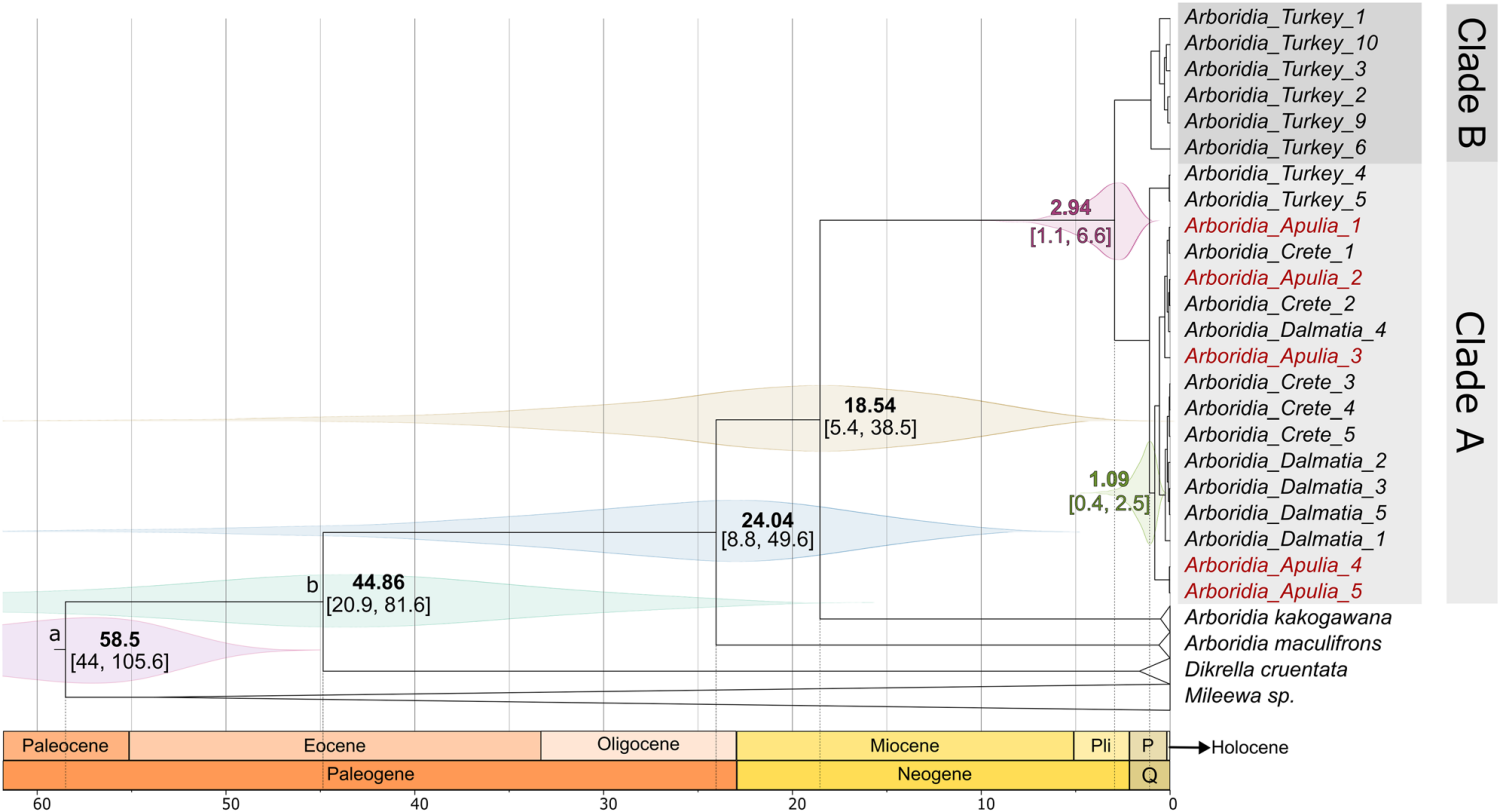
Molecular divergence of *Arboridia* COI gene. The estimated marginal density function is shown on each node. The clock has been calibrated at node a (minimum 44.4 MYA) and b (17.5–90 MYA). Values of x-axis are MYA. Along the x-axis (periods and epochs): P = Pleistocene, Pli = Pliocene, Q = Quaternary.

To calibrate the phylogeny, we utilised sequences from highly divergent taxa, specifically *Mileewa* spp. and *Dikrella cruentata*, and employed DAMBE to assess the presence of substitution saturation; this analysis revealed a negligible to minimal saturation level (P-invariant = 0.219, Iss = 0.565, Iss.c = 0.717, p-value = 0.001). According to the divergence times estimated by a molecular clock analysis of the COI gene (Fig. 7), the radiation of the *A. adanae-dalmatina* clade occurred 2.94 million years ago (95% high posterior density between 1.11 million years ago and 6.59 million years ago) straddling the Pliocene and Pleistocene. Subsequently, Clade A diverged from the Clade B and initiated its current radiation about 1.09 million years ago (95% high posterior density between 386,000 years ago and 2.47 million years ago), in the mid Pleistocene, characterised by alternating glaciation and warming events^44,45^. During the late Pliocene, temperatures decreased leading to the glaciation events that happened during Pleistocene^46^. At this time, Turkey, southern Italy, Dalmatia, and the southern Balkans were glacial refugia^47,48^, areas where species could have survived the more northerly glaciations and then recolonised the surrounding areas following glacial retreat. This suggests that this species might have lived in the eastern Mediterranean area, from the Balkans to Turkey, evolving at the proximity of the ice limit of this region, in a paleoecological scenario characterised by climate conditions that were similar to those of its current distribution. Following the retreat of the glaciers, they enlarged their distribution, but were not able to reach the Apulian peninsula, with only limited genetic divergence between Turkish and Balkanic populations. This scenario is supported by our molecular and morphological data suggesting a species complex or two subspecies rather than two different species. Importantly, from a pest management perspective, with increasing temperatures due to climate change and the geographical conformation of the Italian peninsula, which aids both natural and human movement of the leafhoppers along its coastlines, the invasion and establishment of eastern Mediterranean *Arboridia* is increasingly likely, and represents a potential threat to vineyards and, in general, to the temperate ecosystems of the rest of Italy and of other Mediterranean regions.

### Microbial profiles and presence of Wolbachia

**Figure 8:**
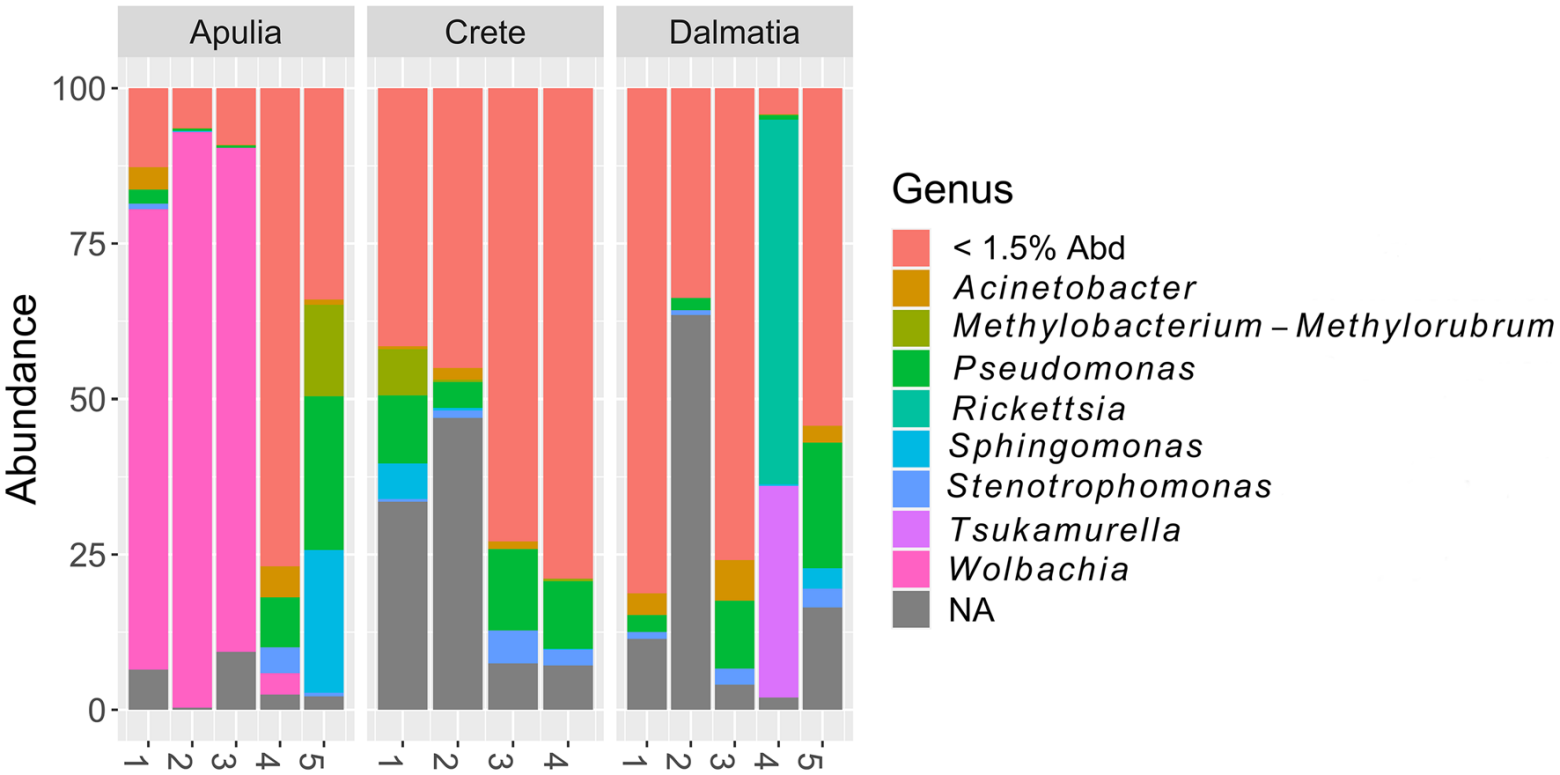
Relative abundance plot of *Arboridia *bacterial microbiota genera (16S V3-V4 region), from the three sampled European locations: Apulia, Crete and Dalmatia. Abd = abundance.

Regarding the microbiota of the Arboridia studied here, the abundance plot (Fig. 8; Table S2) illustrates bacterial genera with a number of reads greater than 1.5% of total reads. Individuals from the three European locations did not differ significantly in terms of alpha diversity (both Chao1 and Shannon indices) at either genus and phylum levels, or in terms of beta diversity at the phylum level (Fig. S1). However, we found beta diversity was significantly different among populations for genera (PERMANOVA R^2^ = 0.28, P = 0.041), although  pairwise differences were only significant between Apulian and Cretan populations (pairwise adonis R^2^ = 0.47, P = 0.024). These results support the hypothesis that the three European populations are not different species and that there is a relatively recent mixing, especially in Apulia, likely due to trades.

It is difficult to assess whether these genera have positive or negative implications on *Arboridia* biology and/or on its management, since pathogenicity or other characteristics of microorganisms are often related to species or strains rather than genera. For example, *Pseudomonas fluorescens* positively affects plants^49^, while *P. syringae* causes diseases in many crops^50^. However, the only genus common among all specimens is *Pseudomonas. Rickettsia* and *Tsukamurella* are present only in the Dalmatian specimen 4, while other abundant genera, except for *Wolbachia*, are widespread among all specimens. However, we were able to identify 270 species of bacteria present in our samples, among which three notable plant pathogens: *Clavibacter michiganensis* in Apulian specimen 5, *Curtobacterium flaccumfaciens* in Cretan specimen 1, and *Xanthomonas citri* in Apulian 5 and in Cretan 2 specimens. *Clavibacter michiganensis* (gram-positive) is known for its pathogenic activity in alfalfa, maize, wheat, and its ability to cause bacterial wilt and canker in tomato^51^, while *Curtobacterium flaccumfaciens* (gram-positive) causes bacterial wilt or tan spot of edible dry beans^52^. *Xanthomonas citri* (gram-negative)causes citrus canker in all commercial citrus varieties^53^. These bacteria have not been associated previously with grapevine diseases, and their primary mode of transmission is through wound infections; therefore, *Arboridia* should pose no greater threat to vineyards than other organisms. Indeed, thus far, the primary causes of the above disease outbreaks have been attributed to infected seeds, transplantation, or the use of contaminated tools^51–53^.

From a management standpoint, the significance of *Wolbachia* in *A. adanae* should be addressed further. *Wolbachia* is a genus of obligate intracellular bacteria found in over 65% of insect species^54^ and plays various critical roles as symbionts within their hosts^55^. Nevertheless, their most notable attribute is their capacity to proliferate within specific insect populations, ultimately instigating reproductive changes that facilitate their own transmission. *Wolbachia* bacteria are maternally-inherited intracellular insect-parasites that can induce different reproductive phenotypes through cytoplasmic incompatibility and other processes^55–59^, and has been intensely studied for its potential as a pest control strategy. *Wolbachia* was found with remarkably high abundance in three Apulian specimens (1, 2, and 3, > 70%) as well as in specimen 4 with a relatively low abundance (< 5%), but it was not found in any of the other individuals sampled outside Italy. Despite the relatively low number of samples processed here, the presence of *Wolbachia* only in the Apulian samples is puzzling. One possibility is that the invasive Italian individuals originate from infected populations that were not sampled for this study. Since the Apulian sampling location has specimens originated from at least two different invasions, one must have been infected by *Wolbachia*. An alternative hypothesis is that *Wolbachia* might have been recently transferred horizontally to *Arboridia* through parasitoids^54–56^ in Italy, after the leafhopper invasion. For example, it is known that parasitoids from Mymaridae and Trichogrammatidae can infect various *Arboridia* species and therefore horizontal transmission of *Wolbachia* might happen^63–65^.

## Conclusion and future perspectives

In this article we characterised an invasive Mediterranean *Arboridia* species by combining results from COI phylogenetics and divergence estimates, with morphological studies and microbiota studies. Our complementary set of results has allowed a first general evaluation of the evolutionary biology of this insect pest, showing the value of a multidisciplinary approach in invasive species research.

We found the first molecular evidence that Turkey and the Balkans may host the same species of *Arboridia*. Through analyses of COI sequences, we observed unlikely phylogenetic relationships between individuals previously identified as *A. adanae* and *A. dalmatina*; in addition, there were no clear morphological differences between individuals from different regions, neither in body size nor in male genitalia. Therefore, we propose to merge *A. adanae* and *A. dalmatina* into a single species, which is *A. adanae* Dlabola, 1957, as a junior synonym of *A. dalmatina* Wagner, 1962.

Phylogenetic analysis also showed that the three genetic clusters of *Arboridia* living on grapevines in the Mediterranean basin were very closely related and geographically heterogeneous. Although this makes it difficult to assess the origin of the Apulian invasion, it suggests that the introduction of this species was a relatively recent event, possibly attributable to human activities. Indeed, the Apulian organic vineyard where we have sampled is located near commercial harbours connected to the Balkans, Greece, and Turkey (approximately 12 km). Overall, because Clade A is composed entirely of Turkish samples, but Turkish samples are also present in Clade B; our phylogenies indicate that Dalmatian and Greek population originated from Turkish population as well. Our clock analysis indicates that these events occurred from the late Pliocene to the Pleistocene. In particular, Dalmatian, Apulian, and Cretan specimens were the result of radiation in the mid Pleistocene, likely in a southern European glacial refugia. This is suggestive of a pre-adaptation to temperate environments and therefore of a predisposition to the current temperate climate of Italy. This discovery holds significant implications from an agricultural standpoint, as *Arboridia* in Apulia has been associated with grapevine chlorosis. Considering the cultural and economic importance of viticulture products in Italy, which fuels a thriving domestic market and international exports across the Mediterranean and beyond, our finding underscores the crucial need to monitor the spread of these leafhoppers throughout the Italian peninsula.

Regarding insights into plant disease transmission, despite the presence of important phytopathogens in a few specimens, *Arboridia* should not pose a greater threat than other insects, since the species is mesophyll feeder and phytopathogens found are known to be transmitted by atmospheric agents and mechanical vectors (e.g. agricultural tools and insects).

Our phylogenies are based on only one mitochondrial marker, mainly due to a lack of molecular data available for *Arboridia* genus. It is well known that there may be discrepancies between mitochondrial and nuclear markers with regards both the genealogy and the divergence estimates^66^. In order to have a more general and reliable picture of *Arboridia* evolution, future analysis should be extended to nuclear genes and more broadly to a genome-scaled dataset including both nuclear and mitochondrial information. Sampling across the Balkans and interbreeding assays should also be added to assess whether *A. adanae* is a species complex or two subspecies.

### Supplementary Information

Supplementary Information.

## Data Availability

All data cited in the manuscript and used for the analyses are available from the corresponding authors on request. *Arboridia* sequences are available on NCBI and their IDs are available in Table S1. The code used for forward and reverse COI sequences assembly is available at https://github.com/RiccPicc/forev. The code used for the barcoding analysis is available at https://github.com/AleTatti/Barcoding-Analysis. The code used for the 16S analysis is available at https://github.com/RiccPicc/Arboridia.
