# Supplementary material for: A multidisciplinary approach to tackling invasive species: barcoding, morphology, and metataxonomy of the leafhopper Arboridia adanae
Source: Sci Rep. 2024 Jan 26;14:2229. doi: 10.1038/s41598-023-49410-9 (PMC10817979; doi:10.1038/s41598-023-49410-9)
Supplement: Supplementary file 1 — Supplementary Information. [file 41598_2023_49410_MOESM1_ESM.pdf]

A multidisciplinary approach in tackling invasive species: barcoding, morphology, and metataxonomy of the leafhopper *Arboridia adanae*

Riccardo Piccinno[1,2,3], Alessia Tatti[1,2,4], Sabina Avosani[5], Giulio Galla[6], Valentina Lazazzara[7], Federico Pedrazzoli[2], Nicola Zadra[6,8], Mirco Rodeghiero[1], Gabrijel Seljak[9], İnanç Özgen[10], Heidi C. Hauffe[6], Vincenzo Verrastro[11], Marco Valerio Rossi Stacconi[2], Valerio Mazzoni[2,*], Omar Rota-Stabelli[1,2,*]

[1] Center Agriculture Food Environment (C3A), University of Trento, San Michele all'Adige, Trento, Italy

[2] Plant Protection Unit, Research and Innovation Centre, Fondazione Edmund Mach, San Michele all'Adige, Trento, Italy

[3] Department of Biology and Biotechnology "L. Spallanzani", University of Pavia, Pavia, Italy

[4] Scuola Universitaria Superiore IUSS Pavia, Pavia, Italy

[5] Faculty of Science and Medicine, University of Fribourg, Fribourg, Switzerland

[6] Conservation Genomics Research Unit, Research and Innovation Centre, Fondazione Edmund Mach, San Michele all'Adige, Trento, Italy

[7] Institute for Sustainable Plant Protection, National Research Council of Italy, Sesto Fiorentino, Florence, Italy

[8] NBFC, National Biodiversity Future Center, Palermo, Italy

[9] Kromberška cesta 8, 5000 Nova Gorica, Slovenia

[10] Bioengineering Department, Engineering Faculty, Fırat University, Elazığ, Turkey

[11] International Centre for Advanced Mediterranean Agronomic Studies (CIHEAM) - Bari, Valenzano, Bari, Italy

* Corresponding authors

Corresponding authors email valerio.mazzoni@fmach.it, omar.rotastabelli@unitn.it

Supplementary information

Alpha and beta diversities of microbial communities

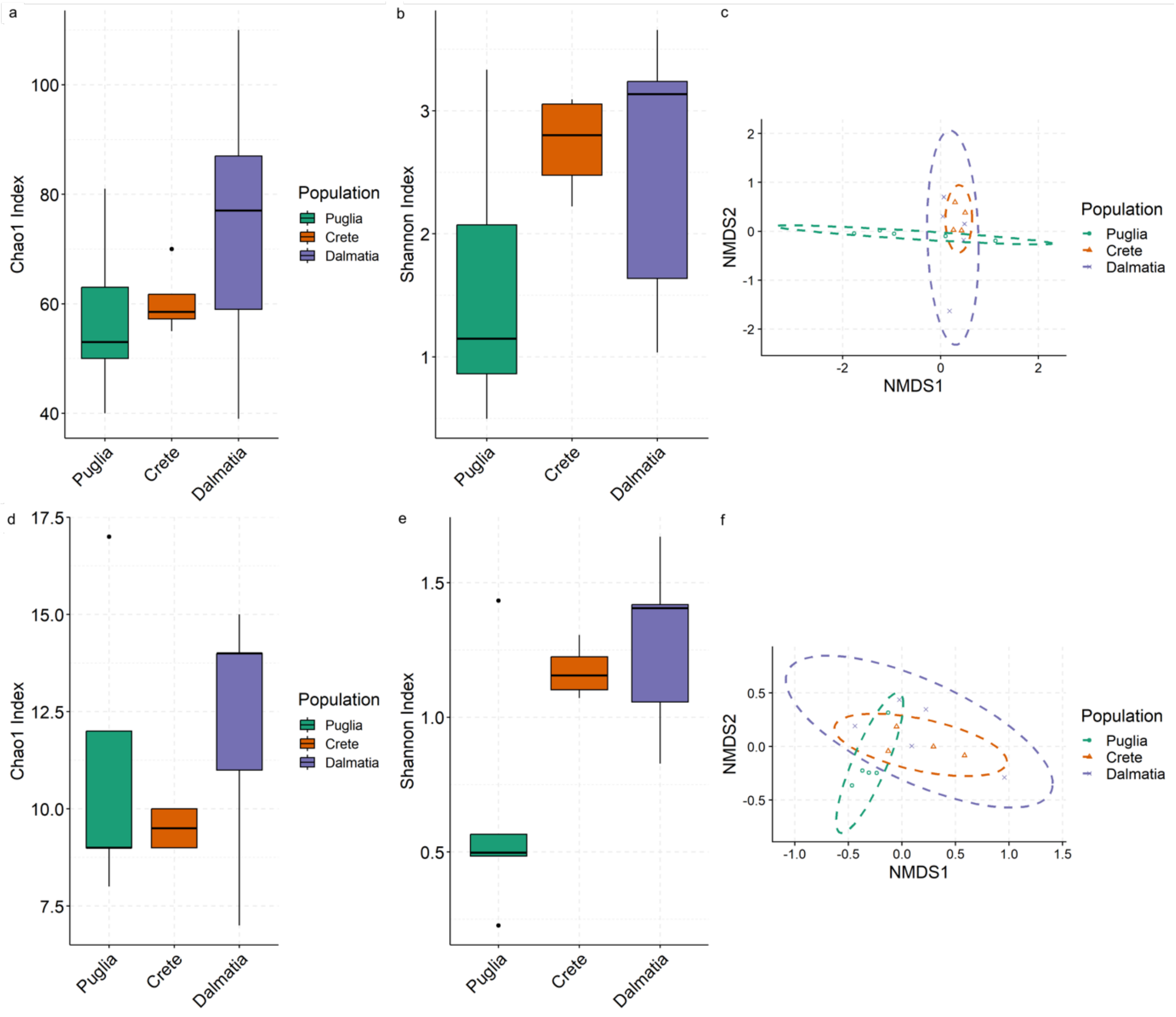

Fig. S1. Plots about alpha and beta diversity of microbiota of Apulian, Cretan and Dalmatian individuals. The three plots on top (**a**, **b** and **c**) are results of the 16S analysis at the genus level. The three plots on top (**d**, **e** and **f**) are results of the 16S analysis at the phylum level. **a**) Chao1 index at the genus level. All comparisons are not significant (n.s.). **b**) Shannon index at the genus level. All

comparisons are n.s. **c**) NMDS plot based on dissimilarities at the genus level (PERMANOVA R^2 = 0.28, P = 0.041). Pairwise adonis n.s. for any contrasts. **d**) Chao1 index at the phylum level. All comparisons are not significant (n.s.). **e**) Shannon index at the phylum level. All comparisons are n.s. **f**) NMDS plot based on dissimilarities at the phylum level (PERMANOVA R^2 = 0.29, P = 0.059). Pairwise adonis significant only for the contrast "Apulia vs. Crete" (R^2 = 0.47, P = 0.024).

COI sequences used for phylogeny

Table S1. Sequences downloaded from NCBI nucleotide to perform the phylogenetic and molecular divergence analysis. Our 23 sequences (5 Apulian, 5 Dalmatian, 5 Cretan and 8 Turkish) were uploaded on NCBI.

**NCBI ID**	**Species**	**NCBI ID**	**Species**
MN972661.1	*Arboridia kakogawana*	MN972656.1	*Arboridia maculifrons*
MN972662.1	*Arboridia kakogawana*	MN972655.1	*Arboridia maculifrons*
MN972665.1	*Arboridia kakogawana*	MN972660.1	*Arboridia maculifrons*
MN972667.1	*Arboridia kakogawana*	MF935009.1	*Dikrella cruentata*
MN972663.1	*Arboridia kakogawana*	MF929911.1	*Dikrella cruentata*
MN972668.1	*Arboridia kakogawana*	MF934624.1	*Dikrella cruentata*
MN972664.1	*Arboridia kakogawana*	MF933368.1	*Dikrella cruentata*
MN972666.1	*Arboridia kakogawana*	MG398285.1	*Dikrella cruentata*
MN972669.1	*Arboridia kakogawana*	MF932414.1	*Dikrella cruentata*
MN972659.1	*Arboridia maculifrons*	MF931840.1	*Dikrella cruentata*
MN972658.1	*Arboridia maculifrons*	MF934830.1	*Dikrella cruentata*
MN972657.1	*Arboridia maculifrons*	MF932997.1	*Dikrella cruentata*
MN972654.1	*Arboridia maculifrons*	KT623269.1	*Dikrella cruentata*

KR581114.1	*Dikrella cruentata*	MK603803.1	*Mileewa sp.*
MK603796.1	*Mileewa sp.*	MK603792.1	*Mileewa sp.*
MK603800.1	*Mileewa sp.*	MK603799.1	*Mileewa sp.*
MK603793.1	*Mileewa sp.*	MK603801.1	*Mileewa sp.*
MK603797.1	*Mileewa sp.*	MK603791.1	*Mileewa sp.*
MK603788.1	*Mileewa sp.*	MK603802.1	*Mileewa sp.*
MK603794.1	*Mileewa sp.*	MK603789.1	*Mileewa sp.*
MK603787.1	*Mileewa sp.*	OQ410656	Apulian *Arboridia sp.*
OQ410659	Apulian *Arboridia sp.*	OQ410661	Apulian *Arboridia sp.*
OQ410669	Apulian *Arboridia sp.*	OQ410670	Apulian *Arboridia sp.*
OQ410658	Cretan *Arboridia sp.*	OQ410657	Cretan *Arboridia sp.*
OQ410662	Cretan *Arboridia sp.*	OQ410663	Cretan *Arboridia sp.*
OQ410664	Cretan *Arboridia sp.*	OQ410668	Dalmatian *Arboridia sp.*
OQ410665	Dalmatian *Arboridia sp.*	OQ410667	Dalmatian *Arboridia sp.*
OQ410660	Dalmatian *Arboridia sp.*	OQ410666	Dalmatian *Arboridia sp.*
OQ410671	Turkish *Arboridia sp.*	OQ410672	Turkish *Arboridia sp.*
OQ410673	Turkish *Arboridia sp.*	OQ410674	Turkish *Arboridia sp.*
OQ410675	Turkish *Arboridia sp.*	OQ410676	Turkish *Arboridia sp.*

OQ410677	Turkish *Arboridia sp.*	OQ410678	Turkish *Arboridia sp.*

Most abundant bacteria detected

Table S2. Twenty most abundant bacterial genus for each specimen.

Population	Specimen	Genus	Population	Specimen	Genus
Apulia	1	*Wolbachia*	Apulia	2	*Staphylococcus*
Apulia	1	*Acinetobacter*	Apulia	2	*Corynebacterium*
Apulia	1	*Pseudomonas*	Apulia	2	*Stenotrophomonas*
Apulia	1	*Anaerobacillus*	Apulia	2	*Bacillus*
Apulia	1	*Burkholderia-Caballeronia-Paraburkholderia*	Apulia	2	*Anaerobacillus*
Apulia	1	*Stenotrophomonas*	Apulia	2	*Cutibacterium*
Apulia	1	*Methylobacterium-Methylorubrum*	Apulia	2	*Craurococcus-Caldovatus*
Apulia	1	*Bacillus*	Apulia	2	*Rickettsia*
Apulia	1	*Cutibacterium*	Apulia	2	*Tsukamurella*
Apulia	1	*Staphylococcus*	Apulia	2	*Methylobacterium-Methylorubrum*
Apulia	1	*Sphingomonas*	Apulia	2	*Veillonella*
Apulia	1	*Craurococcus-Caldovatus*	Apulia	2	*Mycobacterium*
Apulia	1	*Rickettsia*	Apulia	2	*Sphingobacterium*
Apulia	1	*Tsukamurella*	Apulia	2	*Tepidimonas*
Apulia	1	*Corynebacterium*	Apulia	2	*Blastococcus*
Apulia	1	*Veillonella*	Apulia	3	*Wolbachia*
Apulia	1	*Mycobacterium*	Apulia	3	*Bacillus*
Apulia	1	*Sphingobacterium*	Apulia	3	*Pseudomonas*
Apulia	1	*Tepidimonas*	Apulia	3	*Acinetobacter*

Apulia	1	*Blastococcus*	Apulia	3	*Anaerobacillus*
Apulia	2	*Wolbachia*	Apulia	3	*Stenotrophomonas*
Apulia	2	*Pseudomonas*	Apulia	3	*Staphylococcus*
Apulia	2	*Sphingomonas*	Apulia	3	*Cutibacterium*
Apulia	2	*Acinetobacter*	Apulia	3	*Burkholderia-Caballeronia-Paraburkholderia*
Apulia	2	*Burkholderia-Caballeronia-Paraburkholderia*	Apulia	3	*Corynebacterium*
Apulia	3	*Methylobacterium-Methylorubrum*	Apulia	4	*Veillonella*
Apulia	3	*Tepidimonas*	Apulia	4	*Sphingobacterium*
Apulia	3	*Mycobacterium*	Apulia	4	*Tepidimonas*
Apulia	3	*Sphingomonas*	Apulia	4	*Blastococcus*
Apulia	3	*Craurococcus-Caldovatus*	Apulia	5	*Methylobacterium-Methylorubrum*
Apulia	3	*Rickettsia*	Apulia	5	*Pseudomonas*
Apulia	3	*Tsukamurella*	Apulia	5	*Sphingomonas*
Apulia	3	*Veillonella*	Apulia	5	*Acinetobacter*
Apulia	3	*Sphingobacterium*	Apulia	5	*Stenotrophomonas*
Apulia	3	*Blastococcus*	Apulia	5	*Anaerobacillus*
Apulia	4	*Acinetobacter*	Apulia	5	*Corynebacterium*
Apulia	4	*Pseudomonas*	Apulia	5	*Staphylococcus*
Apulia	4	*Bacillus*	Apulia	5	*Burkholderia-Caballeronia-Paraburkholderia*
Apulia	4	*Stenotrophomonas*	Apulia	5	*Bacillus*
Apulia	4	*Wolbachia*	Apulia	5	*Cutibacterium*
Apulia	4	*Cutibacterium*	Apulia	5	*Tepidimonas*

Apulia	4	*Anaerobacillus*	Apulia	5	*Veillonella*
Apulia	4	*Staphylococcus*	Apulia	5	*Wolbachia*
Apulia	4	*Corynebacterium*	Apulia	5	*Craurococcus-Caldovatus*
Apulia	4	*Burkholderia-Caballeronia-Paraburkholderia*	Apulia	5	*Rickettsia*
Apulia	4	*Mycobacterium*	Apulia	5	*Tsukamurella*
Apulia	4	*Methylobacterium-Methylorubrum*	Apulia	5	*Mycobacterium*
Apulia	4	*Sphingomonas*	Apulia	5	*Sphingobacterium*
Apulia	4	*Craurococcus-Caldovatus*	Apulia	5	*Blastococcus*
Apulia	4	*Rickettsia*	Crete	1	*Methylobacterium-Methylorubrum*
Apulia	4	*Tsukamurella*	Crete	1	*Pseudomonas*
Crete	1	*Sphingomonas*	Crete	2	*Corynebacterium*
Crete	1	*Acinetobacter*	Crete	2	*Sphingomonas*
Crete	1	*Burkholderia-Caballeronia-Paraburkholderia*	Crete	2	*Veillonella*
Crete	1	*Staphylococcus*	Crete	2	*Staphylococcus*
Crete	1	*Cutibacterium*	Crete	2	*Tepidimonas*
Crete	1	*Bacillus*	Crete	2	*Blastococcus*
Crete	1	*Stenotrophomonas*	Crete	2	*Wolbachia*
Crete	1	*Anaerobacillus*	Crete	2	*Craurococcus-Caldovatus*
Crete	1	*Blastococcus*	Crete	2	*Rickettsia*
Crete	1	*Veillonella*	Crete	2	*Tsukamurella*
Crete	1	*Corynebacterium*	Crete	2	*Mycobacterium*
Crete	1	*Wolbachia*	Crete	2	*Sphingobacterium*

Crete	1	*Craurococcus-Caldovatus*	Crete	3	*Acinetobacter*
Crete	1	*Rickettsia*	Crete	3	*Pseudomonas*
Crete	1	*Tsukamurella*	Crete	3	*Burkholderia-Caballeronia-Paraburkholderia*
Crete	1	*Mycobacterium*	Crete	3	*Stenotrophomonas*
Crete	1	*Sphingobacterium*	Crete	3	*Bacillus*
Crete	1	*Tepidimonas*	Crete	3	*Anaerobacillus*
Crete	2	*Pseudomonas*	Crete	3	*Tepidimonas*
Crete	2	*Acinetobacter*	Crete	3	*Cutibacterium*
Crete	2	*Methylobacterium-Methylorubrum*	Crete	3	*Corynebacterium*
Crete	2	*Burkholderia-Caballeronia-Paraburkholderia*	Crete	3	*Methylobacterium-Methylorubrum*
Crete	2	*Stenotrophomonas*	Crete	3	*Staphylococcus*
Crete	2	*Anaerobacillus*	Crete	3	*Wolbachia*
Crete	2	*Bacillus*	Crete	3	*Sphingomonas*
Crete	2	*Cutibacterium*	Crete	3	*Craurococcus-Caldovatus*
Crete	3	*Rickettsia*	Dalmatia	1	*Blastococcus*
Crete	3	*Tsukamurella*	Dalmatia	1	*Sphingobacterium*
Crete	3	*Veillonella*	Dalmatia	1	*Acinetobacter*
Crete	3	*Mycobacterium*	Dalmatia	1	*Mycobacterium*
Crete	3	*Sphingobacterium*	Dalmatia	1	*Pseudomonas*
Crete	3	*Blastococcus*	Dalmatia	1	*Craurococcus-Caldovatus*
Crete	4	*Pseudomonas*	Dalmatia	1	*Anaerobacillus*
Crete	4	*Burkholderia-Caballeronia-*	Dalmatia	1	*Staphylococcus*

		Paraburkholderia			
Crete	4	*Bacillus*	Dalmatia	1	*Stenotrophomonas*
Crete	4	*Staphylococcus*	Dalmatia	1	*Sphingomonas*
Crete	4	*Anaerobacillus*	Dalmatia	1	*Bacillus*
Crete	4	*Stenotrophomonas*	Dalmatia	1	*Burkholderia-Caballeronia-Paraburkholderia*
Crete	4	*Acinetobacter*	Dalmatia	1	*Cutibacterium*
Crete	4	*Corynebacterium*	Dalmatia	1	*Corynebacterium*
Crete	4	*Cutibacterium*	Dalmatia	1	*Veillonella*
Crete	4	*Sphingomonas*	Dalmatia	1	*Methylobacterium-Methylorubrum*
Crete	4	*Methylobacterium-Methylorubrum*	Dalmatia	1	*Wolbachia*
Crete	4	*Mycobacterium*	Dalmatia	1	*Rickettsia*
Crete	4	*Wolbachia*	Dalmatia	1	*Tsukamurella*
Crete	4	*Craurococcus-Caldovatus*	Dalmatia	1	*Tepidimonas*
Crete	4	*Rickettsia*	Dalmatia	2	*Staphylococcus*
Crete	4	*Tsukamurella*	Dalmatia	2	*Pseudomonas*
Crete	4	*Veillonella*	Dalmatia	2	*Burkholderia-Caballeronia-Paraburkholderia*
Crete	4	*Sphingobacterium*	Dalmatia	2	*Anaerobacillus*
Crete	4	*Tepidimonas*	Dalmatia	2	*Bacillus*
Crete	4	*Blastococcus*	Dalmatia	2	*Cutibacterium*
Dalmatia	2	*Stenotrophomonas*	Dalmatia	3	*Methylobacterium-Methylorubrum*
Dalmatia	2	*Acinetobacter*	Dalmatia	3	*Wolbachia*
Dalmatia	2	*Sphingomonas*	Dalmatia	3	*Rickettsia*

Dalmatia	2	*Methylobacterium-Methylorubrum*
Dalmatia	2	*Corynebacterium*
Dalmatia	2	*Blastococcus*
Dalmatia	2	*Wolbachia*
Dalmatia	2	*Craurococcus-Caldovatus*
Dalmatia	2	*Rickettsia*
Dalmatia	2	*Tsukamurella*
Dalmatia	2	*Veillonella*
Dalmatia	2	*Mycobacterium*
Dalmatia	2	*Sphingobacterium*
Dalmatia	2	*Tepidimonas*
Dalmatia	3	*Staphylococcus*
Dalmatia	3	*Pseudomonas*
Dalmatia	3	*Acinetobacter*
Dalmatia	3	*Veillonella*
Dalmatia	3	*Stenotrophomonas*
Dalmatia	3	*Anaerobacillus*
Dalmatia	3	*Tepidimonas*
Dalmatia	3	*Burkholderia-Caballeronia-Paraburkholderia*
Dalmatia	3	*Craurococcus-Caldovatus*
Dalmatia	3	*Cutibacterium*
Dalmatia	3	*Sphingomonas*
Dalmatia	3	*Tsukamurella*
Dalmatia	3	*Bacillus*
Dalmatia	3	*Mycobacterium*
Dalmatia	3	*Sphingobacterium*
Dalmatia	3	*Blastococcus*
Dalmatia	4	*Rickettsia*
Dalmatia	4	*Tsukamurella*
Dalmatia	4	*Staphylococcus*
Dalmatia	4	*Pseudomonas*
Dalmatia	4	*Sphingomonas*
Dalmatia	4	*Burkholderia-Caballeronia-Paraburkholderia*
Dalmatia	4	*Acinetobacter*
Dalmatia	4	*Anaerobacillus*
Dalmatia	4	*Bacillus*
Dalmatia	4	*Stenotrophomonas*
Dalmatia	4	*Cutibacterium*
Dalmatia	4	*Wolbachia*
Dalmatia	4	*Craurococcus-Caldovatus*
Dalmatia	4	*Methylobacterium-Methylorubrum*
Dalmatia	4	*Corynebacterium*
Dalmatia	4	*Veillonella*
Dalmatia	4	*Mycobacterium*

Dalmatia	3	*Corynebacterium*	Dalmatia	4	*Sphingobacterium*
Dalmatia	4	*Tepidimonas*	Dalmatia	5	*Bacillus*
Dalmatia	4	*Blastococcus*	Dalmatia	5	*Methylobacterium-Methylorubrum*
Dalmatia	5	*Pseudomonas*	Dalmatia	5	*Tsukamurella*
Dalmatia	5	*Sphingomonas*	Dalmatia	5	*Wolbachia*
Dalmatia	5	*Acinetobacter*	Dalmatia	5	*Craurococcus-Caldovatus*
Dalmatia	5	*Staphylococcus*	Dalmatia	5	*Rickettsia*
Dalmatia	5	*Stenotrophomonas*	Dalmatia	5	*Corynebacterium*
Dalmatia	5	*Anaerobacillus*	Dalmatia	5	*Veillonella*
Dalmatia	5	*Burkholderia-Caballeronia-Paraburkholderia*	Dalmatia	5	*Mycobacterium*
Dalmatia	5	*Tepidimonas*	Dalmatia	5	*Sphingobacterium*
Dalmatia	5	*Cutibacterium*	Dalmatia	5	*Blastococcus*
